# Theanine Improves Salt Stress Tolerance *via* Modulating Redox Homeostasis in Tea Plants (*Camellia sinensis* L.)

**DOI:** 10.3389/fpls.2021.770398

**Published:** 2021-10-15

**Authors:** Ziping Chen, Shijia Lin, Juan Li, Tingting Chen, Quan Gu, Tianyuan Yang, Zhaoliang Zhang

**Affiliations:** ^1^State Key Laboratory of Tea Plant Biology and Utilization, Anhui Agricultural University, Hefei, China; ^2^Biotechnology Center, Anhui Agricultural University, Hefei, China; ^3^School of Biology, Food and Environment, Hefei University, Hefei, China

**Keywords:** *Camellia sinensis* L., salt stress, theanine biosynthesis, ROS scavenging enzymes, catalases

## Abstract

Theanine, a unique non-proteinogenic amino acid, is one of the most abundant secondary metabolites in tea. Its content largely determines green tea quality and price. However, its physiological roles in tea plants remain largely unknown. Here, we showed that salt stress significantly increased the accumulation of glutamate, glutamine, alanine, proline, and γ-aminobutyric acid, as well as theanine, in the new shoots of tea plants. We further found that salt stress induced the expression of theanine biosynthetic genes, including *CsGOGATs*, *CsAlaDC*, and *CsTSI*, suggested that salt stress induced theanine biosynthesis. Importantly, applying theanine to the new shoots significantly enhanced the salt stress tolerance. Similar effects were also found in a model plant Arabidopsis. Notably, exogenous theanine application increased the antioxidant activity of the shoots under salt stress, suggested by reduced the reactive oxygen species accumulation and lipid peroxidation, as well as by the increased SOD, CAT, and APX activities and expression of the corresponding genes. Finally, genetic evidence supported that catalase-mediated antioxidant scavenging pathway is required for theanine-induced salt stress tolerance. Taken together, this study suggested that salt stress induces theanine biosynthesize in tea plants to enhance the salt stress tolerance through a CAT-dependent redox homeostasis pathway.

## Introduction

The tea plant (*Camellia sinensis* L.) is an important economic woody crop, and widely cultivated in the world (Chen et al., 2007). It contains numerous secondary metabolites, including theanine (γ-glutamylethylamide), flavonoids and caffeine. As a unique non-proteinogenic amino acid in tea plants, theanine is the component conferring the particular “umami” tastes, anti-depression and neuroprotection benefits of green tea infusion (Ashihara, 2015). Theanine is the most abundant free amino acid in tea plants, however, its physiological roles in tea plants remains unknown.

It is synthesized from glutamate and ethylamine *via* theanine synthatase (CsTS). In recent study, the TS gene (*CsTSI*) was identified in tea plants (Wei et al., 2018). CsTSI is highly homologous to glutamine synthetases (GSs) which also can catalyze theanine synthesis (Cheng et al., 2017; Wei et al., 2018). Glutamate Synthases (GOGATs) catalyze glutamate biosynthesis in tea plants, while ethylamine is synthesized by alanine decarboxylase (CsAlaDC; Takeo, 1979; Bai et al., 2019). Genes involved in theanine metabolism have been systemically identified along with the completion of the genome sequencing in tea plants (Xia et al., 2019). Theanine metabolism is regulated by many environment factors, especially high salinity, heat, drought, nutrient levels and light intensity (Deng et al., 2012; Wang et al., 2016; Li et al., 2018a; Yang et al., 2020, 2021). However, theanine, as a species-specific secondary metabolite, its roles in the adaption to these abiotic stresses for tea plants need to be explored.

Excessive irrigation and fertilization increase salt contents in soils, which imposes salt stress to crops, including tea plants (Causapé et al., 2004). Salt stress reduces the yield and quality of tea and greatly diminishes the widespread of tea plants (Upadhyaya and Panda, 2013). High soil salinity has become one of the major adverse environmental stresses in global agricultural production. Salt stress usually reduces efficiency of photosynthetic apparatus including PSII and the electron transport chain, and elicits the accumulation of reactive oxygen species (ROS), and ultimately leads to cell damage and oxidative stress (Wang et al., 2007; Considine and Foyer, 2021).

Plants have evolved a complex antioxidant defense system to balance the ROS levels in response to salt stress (Bose et al., 2013). Generally, the superoxide $\left({\text{O}}_{2}^{-}\right)$ is converted into hydrogen peroxide (H_2_O_2_) through superoxide dismutase (SOD). Then, that H_2_O_2_ is converted into H_2_O and oxygen by ascorbate peroxidases (APXs), catalases (CATs), as well as other free radical scavengers (Bose et al., 2013). ROS imbalance is harmful to plants; however, H_2_O_2_ is also involved in the chloroplast-to-nucleus retrograde signaling which is critical for salt stress response in plants (Park and Seo, 2021; Zhuang et al., 2021).

Recent studies have reported that salt stress affects nitrogen (N) and amino acid metabolism in plants (Silveira et al., 2003; Dluzniewska et al., 2007). Ammonium is assimilated first into the glutamine by GSs, and then is assimilated into glutamate by GOGATs in plants (Groat and Vance, 1981; Miflin and Habash, 2002). Glutamate is further used to synthesize other amino acids like proline, γ-aminobutyric acid (GABA) and asparagine, etc. (Yang et al., 2020). Salt stress induces the accumulation of proline and GABA which act as compatible osmolyte or signaling molecule involved in mitigating high salinity stress (Szabados and Savouré, 2010; Hayat et al., 2012; Bao et al., 2015).

In animals, theanine is involved in various physiological functions, such as improvement of sleep quality, relaxation, neuroprotection, and Parkinson’s disease, etc. (Narukawa et al., 2008; Sharma et al., 2018; Deb et al., 2019). Impressively, theanine alleviates Cd-induced oxidative damage through reducing malondialdehyde (MDA) and ROS levels in the brain of mouse (Ben et al., 2016). It was reported that salt stress induces theanine biosynthesis in tea plants (Deng et al., 2012). In addition, exogenous supply of glutamate alleviated the salinity stress-induced inhibition of seed germination and radicle growth of buck wheat and cucumber (Chang et al., 2010; Yang, 2014). Given that theanine is a natural analog of glutamate, we hypothesize that theanine improves salt stress tolerance *via* modulating ROS homeostasis in tea plants.

In this study, we observed that salt stress induced theanine biosynthesis in the new shoots of tea plants, and exogenous theanine application enhanced the resistance to salt stress in the new shoots. We explored the underlying mechanism, with particular emphasis on the reestablishment of redox homeostasis by theanine. The study provided novel insights into the physiological roles of theanine in tea plants.

## Materials and Methods

### Plant Materials and Treatments

New shoots were collected from tea plants (*Camellia sinensis* var. *sinensis cv.* “Shuchazao”) grown in the tea plantation of Anhui Agricultural University in Hefei, China. For salt stress treatment, new shoots were exposed to nutrient solution containing 0, 100, 150, or 200 mM NaCl. To study the function of theanine, new shoots were exposed to nutrient solution containing 150 mM NaCl (NaCl), 10 mM theanine (Thea), 150 mM NaCl and 10 mM Thea (NaCl + Thea). Control samples (Con) were exposed to normal nutrient solution. The composition of the nutrient solution used was as follows: 0.54 mM (NH_4_)_2_SO_4_, 0.18 mM Ca(NO_3_)_2_, 0.1 mM, KH_2_PO_4_, 0.41 mM K_2_SO_4_, 0.39 mM CaCl_2_, 1.03 mM MgSO_4_, 6.27 μM C_10_H_12_FeN_2_NaO_8_, 9.25 μM H_3_BO_3_, 3.9 μM CuSO_4_, 18.2 μM MnSO_4_, 0.4 mM Al_2_(SO_4_)_3_^⋅^18H_2_O, 0.53 μM Na_2_MoO_4_, and 1.53 μM ZnSO_4_, and the pH of the nutrient solution was adjusted to 4.5–5.0 (Konishi et al., 1985).

Arabidopsis seeds of *cat2cat3* and *cat1cat2cat3* mutants were generous gifts from Professor Changle Ma in Shandong Normal University, China. Arabidopsis seeds were surface-sterilized by sodium hypochlorite and rinsed four times with sterile water, and then cultured on the solid Murashige and Skoog (MS) medium (pH 5.8) containing 1% (w/v) agar and 1% (w/v) sucrose. Seeds were stratified at 4°C for 2 days, and then transferred into the growth chamber with day/night cycle of 16 h/8 h, 150 μmol m^–2^ s^–1^ irradiance, 23/18°C, and 70% relative humidity. Arabidopsis wild-type (WT) and mutants were grown on MS medium containing 0 or 100 mM NaCl with or without 1 mM theanine. The time points of treatments were illustrated in the corresponding legends. After treatments, seedlings were imaged using a Canon IXUS 130 camera. Meanwhile, all tissues were sampled according to the demands of each experiment, and immediately frozen in liquid nitrogen and stored at −80°C until utilization.

### Determination of Fv/Fm

After treatments, the new shoots of tea plants were dark adapted for 30 min before measurement of chlorophyll fluorescence. Maximum photochemical efficiency of photosystem II (Fv/Fm) was measured in the 2nd leaves by an imaging-PAM chlorophyll fluorimeter fitted with a computer-operated PAM-control unit (IMAG-MAXI; Heinz Walz, Effeltrich, Germany) as described previously (Li et al., 2018b).

### Determination of Chlorophyll Content

Total chlorophyll in seedlings leaves was extracted using 95% (v/v) ethanol for 48 h in darkness, and the content was then calculated by examining the absorbance at 649, 665, and 470 nm as previously described (Lichtenthaler, 1987).

### Measurement of Free Amino Acids

Amino acids in tea leaves were detected by the method previously described (Lu et al., 2019). Briefly, amino acids were extracted from 0.1 g samples with 2 ml of 4% (w/v) sulfosalicylic acid. After ultrasound extraction for 30 min at 50°C, and centrifuged at 12,000 *g* for 30 min, supernatants were filtered through a 0.22 μm filter for the amino acid content assay. Amino acids were separated by the High-Speed Amino Acid Analyzer system used a mobile phase involving lithium citrate and UV–Vis detection at 570 and 440 nm. The amounts of amino acids were determined according to the calibration curve of amino acid standard.

### Determination of Theanine Content

Theanine was extracted with distilled water as previously described, with some modifications (Tsushida and Takeo, 1984). About 100 mg of freeze-dried sample powder were dissolved in 3 ml distilled water and heated in a water bath at 100°C for 30 min. After centrifugation at 13,000 rpm for 20 min, the supernatant was filtered with 0.22 μm filter for subsequent HPLC-based analysis of theanine content.

The detection conditions of HPLC analysis were as previously described (Dong et al., 2020). Theanine content was detected using the HPLC analysis (Waters e2695 system consisting a 2489 ultraviolet (UV)-visible detector, Waters Corporation, Milford, MA, United States), equipped with a C18 column (5 μm, 250 mm × 4.6 mm) at 28°C. The mobile phase comprised water **(A)**, acetonitrile **(B)**, and the gradient elution was performed as follows: B 0% (v/v) to 100% at 40 min, to 100% at 45 min and to 0% at 60 min. The amount of theanine was determined according to a calibration curve of theanine standard. L-Theanine standard and acetonitrile were purchased from Sigma-Aldrich (St Louis, MO, United States).

### Determination of Reactive Oxygen Species

H_2_O_2_ and superoxide anion $\left({\text{O}}_{2}^{-•}\right)$ levels were detected by histochemical staining methods (Xie et al., 2012). For H_2_O_2_ detection, leaves were incubated in 1 mg ml^–1^ solution of 3, 3′-diaminobenzidine (DAB) in 50 mM Tris-acetate buffer (pH 6.5) for 24 h in darkness at room temperature. For ${\text{O}}_{2}^{-•}$ detection, leaves were immersed in a 0.1% solution of nitroblue tetrazolium (NBT) in K-phosphate buffer (pH 6.5) in darkness for 12 h at room temperature. Afterward, the chlorophyll of leaves was removed with 95% ethanol. All samples were observed using a Canon IXUS 130 camera.

Content of thiobarbituric acid-reactive substance (TBARS; an indicator of lipid peroxidation) was measured by the method previously described (Buege and Aust, 1978). 0.5 g fresh tissue was ground with 3 ml 5% trichloroacetic acid (TCA) using a mortar and pestle, then added with 3 ml 0.5% 2-thiobarbituric acid (TBA) in 5% TCA. After incubation in 100°C water bath for 30 min, centrifuged at 12,000*g* for 15 min, then determined by examining the absorbance at 450, 532, and 600 nm. The concentration of lipid peroxides, together with oxidation modified proteins, was quantified in terms of TBARS amount using an extinction coefficient of 155 mM^–1^ cm^–1^ and expressed as nmol g^–1^ fresh weight (FW).

### Determination of Antioxidant Enzyme

About 0.1 g the 2nd leaf sample was homogenized with 1 ml of 50 mM phosphate buffer (pH 7.8), or together with 1 mM ascorbic acid (ASC; in the case of ascorbate peroxidase activity determination), then centrifuged at 12,000*g* (4°C) for 15 min. Then, supernatants were used for determination of protein concentrations and the enzyme activity assays.

Superoxide dismutase activity was determined by the capacity of inhibiting the photochemical reduction of NBT at A_560_ (Beauchamp and Fridovich, 1971). Catalase (CAT) activity was measured by the absorbance decrease at 240 nm due to the H_2_O_2_ decomposition (extinction coefficient of 40 M^–1^ cm^–1^) (Cui et al., 2013). Ascorbate peroxidase (APX) activity was measured by monitoring the decrease at A_290_ as reduced ascorbic acid was oxidized (extinction coefficient of 2.8 mM^–1^ cm^–1^) (Xie et al., 2008). Protein was determined by the method of Bradford (1976).

### RNA Extraction and Quantitative Real-Time RT-PCR

Total RNA was extracted using an RNAprep Pure kit (Tiangen, Beijing, China), according to the manufacturer’s instructions. RNA concentration and quality were detected by the NanoDrop 2000 (Thermo Fisher Scientific, Wilmington, DE, United States). cDNAs were synthesized from 1 μg of total RNA using an oligo(dT) primer and an EasyScript All-in-One First-Strand cDNA Synthesis SuperMix (One-Step gDNA Removal) Synthesis (TransGen Biotech, Beijing, China). Real-time quantitative reverse-transcription (RT) PCR was conducted using a QuantStudio 6 Flex System (Thermo Lifetech, United States) with TransStart^®^ Green qPCR SuperMix (TransGen Biotech, Beijing, China) by the gene-specific primers (Supplementary Table 1). The expression levels of genes were normalized to *CsGAPDH* transcript level, and presented as values relative to corresponding control samples.

### Statistical Analysis

Statistical analysis was performed using SPSS 18.0 software. Data were means ± SE of at least three biological replicates. The asterisks indicate significant differences by Student’s *t*-test (^∗^*P* < 0.05). For statistical analysis, data was analyzed by one-way analysis of variance (ANOVA) followed by Duncan’s multiple range test, and *P* < 0.05 were considered statistically significant.

## Results

### New Shoots of Tea Plants Were Sensitive to Salt Stress

**FIGURE 1:**
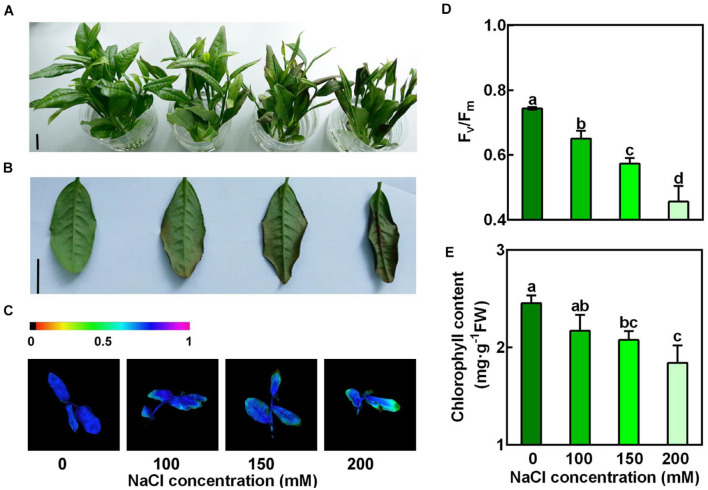
Concentration-dependent effects of NaCl on the new shoots of tea plants. Tea new shoots of tea plants were treated in nutrient solution containing 0, 100, 150, or 200 mM NaCl. **(A,B)** Phenotypes of the new shoots and the 2nd leaves exposed to 0, 100, 150, or 200 mM NaCl treatments. Photographs were taken 3 days after treatment. Bar = 2 cm. **(C,D)** Pseudo-color image and statistical analysis of Fv/Fm of the 2nd leaves 2 days after treatment. The Pseudo-color gradient shows the degree of damage: 1, no damage; 0, strong damage. **(E)** Chlorophyll contents of the 2nd leaves. The contents were determined 3 days after treatment. Data are means ± SE of at least three biological replicates. Bars with different letters are significantly different at *P* < 0.05 according to Duncan’s multiple range test.

To examine the sensitivity of new shoots of tea plants to salt stress, the new shoots sampled from tea plantation were incubated in solutions containing 0, 100, 150, and 200 mM NaCl for 3 days (Figures 1A,B). We observed that 100, 150, and 200 mM NaCl severely damaged these new shoots. The effects of salt stress on the representative physiological characters, such as maximum photochemical efficiency of photosystem II (Fv/Fm) and loss of chlorophyll, were investigated. In our experimental conditions, salt stress significantly lowered the Fv/Fm in the 2nd leaves of the new shoots, in proportion to the NaCl concentrations (Figures 1C,D). Compared to the control, chlorophyll contents were also significantly decreased in high salinity-stressed leaves (Figure 1E). When subjected to 150 and 200 mM NaCl, chlorophyll contents were reduced by 15.5% and 24.9%, respectively. These results indicated high salinity imposed significant damage to the new shoots of tea plants.

### Salt Stress Increased the Accumulation of Glutamate, Glutamine, Proline, γ-Aminobutyric Acid, Alanine and Theanine in the New Shoots

**FIGURE 2:**
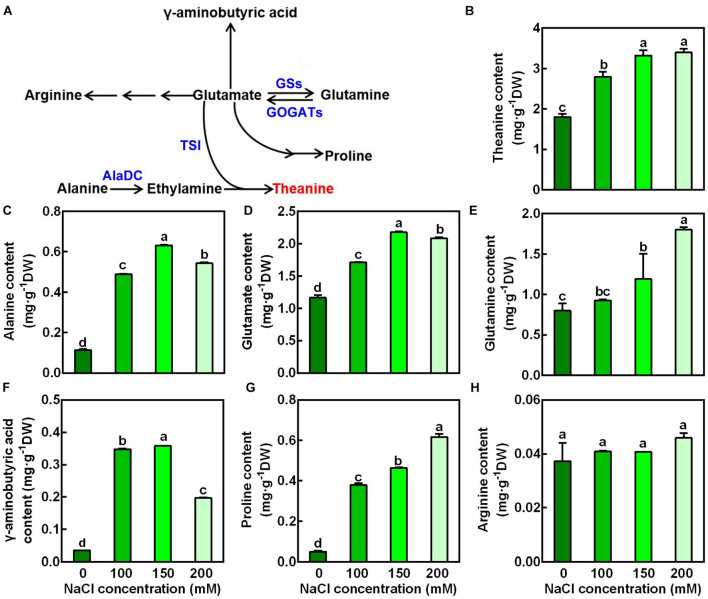
Effects of NaCl treatments on the accumulation of glutamate-pathway and theanine-related amino acids in the 2nd leaves of the new shoots. **(A)** Biosynthetic pathways of glutamate-pathway and theanine-related amino acids. GS, GOGAT, AlaDC, and TSI represent genes encoding glutamine synthetase, glutamate synthase, alanine decarboxylase, and theanine synthatase, respectively. **(B–H)** Amino acid contents in the 2nd leaves of new shoots. The new shoots were treated in nutrient solution containing 0, 100, 150, or 200 mM NaCl for 3 days before the leaves were collected for amino acid content measurement. Theanine **(B)**, alanine **(C)**, glutamate **(D)**, glutamine **(E)**, γ-aminobutyric acid **(F)**, proline **(G)**, arginine **(H)** were detected by the High-Speed Amino Acid Analyzer system. Data are means ± SE of three biological replicates. Bars with different letters are significantly different at *P* < 0.05 according to Duncan’s multiple range test.

To examine the changes of amino acid accumulation in the new shoots in response to salt stress, the 2nd leaves were sampled 3 days after treatment and were used for amino acid measurement by High-Speed Amino Acid Analyzer system. The glutamate pathway amino acids (glutamine, proline, GABA, arginine, and theanine) were then analyzed (Figure 2A). Alanine, which provides ethylamine for theanine synthesis, was also analyzed. As shown in Figure 2B, NaCl treatments significant increased the accumulation of theanine in the new shoots. For example, it was increased by 0.84-fold after 3 days exposure to 150 mM NaCl compared to the control (0 mM NaCl). Besides, it was evident that NaCl treatments markedly increased the accumulation of alanine, glutamate, glutamine, GABA and proline (Figures 2C–G). Glutamate and alanine were increased 0.87- and 4.73-fold by 150 mM NaCl, respectively. In contrast, arginine content was not significantly changed by salt stress (Figure 2H). These results indicated that glutamate pathway amino acids were induced by salt stress, and implied a role of these amino acids in tolerance to salt stress.

**FIGURE 3:**
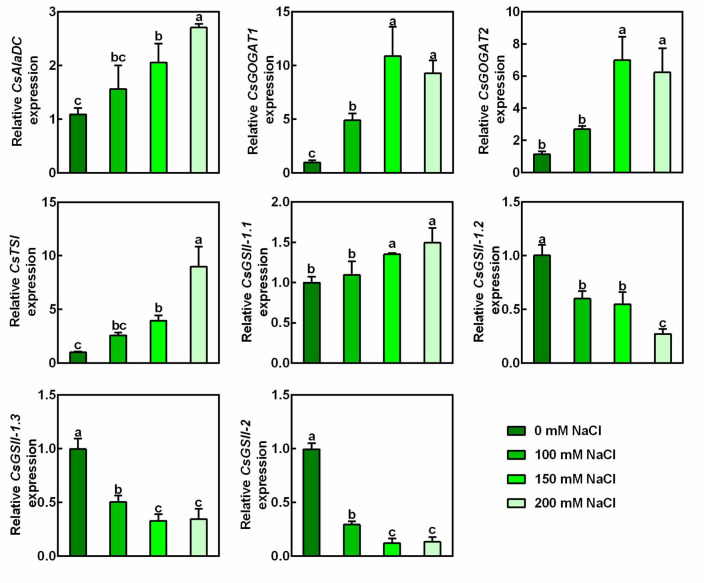
Effects of NaCl treatments on expression of theanine biosynthetic genes in the 2nd leaves of the new shoots. The new shoots of tea plants were treated in nutrient solution containing 0, 100, 150, or 200 mM NaCl. The expression of *CsAlaDC* (TEA005658), *CsGOGAT1* (TEA003892), *CsGOGAT2* (TEA026779), *CsTSI* (TEA015198), *CsGSII-1.1* (TEA015580), *CsGSII-1.2* (TEA032123), *CsGSII-1.3* (TEA032217), and *CsGSII-2* (TEA028194) in the 2nd leaves 2 days after treatment was analyzed by qRT-PCR. Expression levels were presented as values relative to control samples, after normalization to the internal control gene *CsGAPDH* (TEA025584). Data are means ± SE of three biological replicates. Bars with different letters are significantly different at *P* < 0.05 according to Duncan’s multiple range test.

To ascertain the effect of salt stress on the theanine biosynthesis, transcript levels of genes encoding theanine synthatase (TS), alanine decarboxylase (AlaDC), glutamine synthetase (GS), and glutamate synthase (GOGAT) (Figure 2A) were analyzed by real-time RT-PCR. Interestingly, compared to the control condition, the expression of theanine biosynthetic pathway genes, including *CsAlaDC*, *CsGOGAT1*, *CsGOGAT2*, and *CsTSI* was significantly induced by salt stress in a concentration-dependent manner (Figure 3). The expression of *CsGSII-1.1* was also slightly induced; however, the expression of *CsGSII-1.2*, *CsGSII-1.2*, and *CsGSII-2* was largely repressed by salt stress. These results implied salt stress increased theanine accumulation was probably through induced theanine biosynthesis in the new shoots of tea plants.

Given that 150 mM NaCl significantly inhibited Fv/Fm and chlorophyll accumulation, and induced theanine biosynthesis, this concentration of NaCl was chosen for the further experiments.

### Exogenous Theanine Application Alleviated Salt Stress Damage to the New Shoots of Tea Plants

**FIGURE 4:**
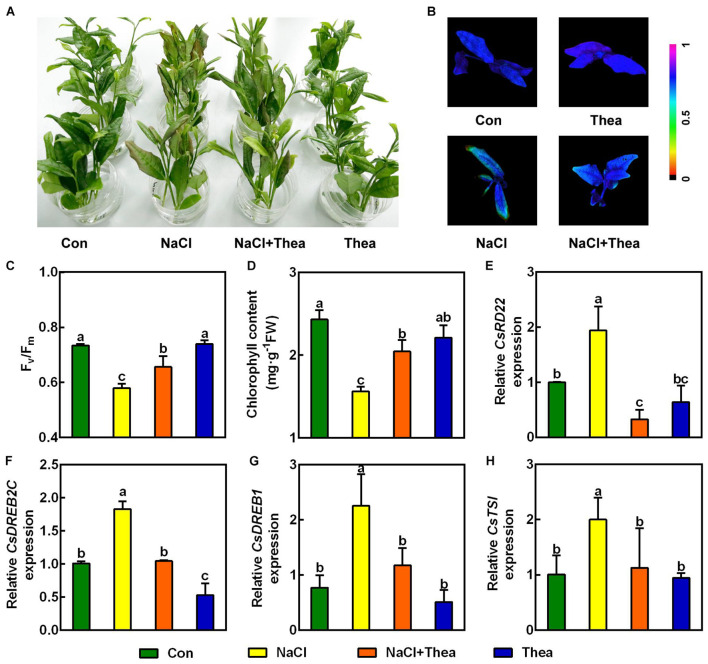
Exogenous theanine alleviated salt stress to the new shoots of tea plants. The new shoots of tea plants were treated in nutrient solution containing 150 mM NaCl (NaCl), 10 mM theanine (Thea), 150 mM NaCl and 10 mM Thea (NaCl + Thea), with normal nutrient solution as control (Con). **(A)** Phenotypes of the new shoots. Photographs were taken 3 days after treatment. **(B,C)** Pseudo-color image and statistical analysis of Fv/Fm of the 2nd leaves 2 days after treatment. The Pseudo-color gradient shows the degree of damage: 1, no damage; 0, strong damage. **(D)** Chlorophyll contents in the 2nd leaves. The contents were determined 3 days after treatment. **(E–H)** The expression of *CsRD22* (TEA005584), *CsDREB2C* (TEA000861), *CsDREB1* (TEA010806), and *CsTSI* (TEA015198) in 2nd leaves. The expression was analyzed 2 days after treatment by qRT-PCR. Expression levels were presented as values relative to Con samples, after normalization to the internal control gene *CsGAPDH* (TEA025584). Data are means ± SE of three biological replicates. Bars with different letters are significantly different at *P* < 0.05 according to Duncan’s multiple range test.

Previously studies showed salt stress-induced proline and GABA improve tolerance to salt stress in plants (Moukhtari et al., 2020; Wu et al., 2020). To examine whether theanine plays a role in enhancing salt stress tolerance in the new shoots of tea plants, the effects of 10 mM exogenous theanine on the salt stress sensitivity, Fv/Fm, and chlorophyll level in the absence and presence of 150 mM NaCl treatment were investigated. Under salt stress treatment, exogenous theanine application significantly alleviated the damage of salt stress to the new shoots. This is evidenced by that exogenous theanine improved the performance, Fv/Fm and chlorophyll content of the new shoots in response to salt stress (Figures 4A–D). In the molecular level, the expression of salt stress-responsive genes, such as *CsRD22*, *CsDREB2C*, *CsDREB1*, and *CsTSI*, was reduced to the control level by theanine (Figures 4 E–H). In addition, application of 1 mM theanine to the model plant Arabidopsis also improved the salt stress tolerance of the seedlings (Supplementary Figure 1). It is noteworthy that exogenous theanine application under normal condition did not showed obvious effects on the new shoots of tea plants and Arabidopsis seedlings (Figures 4A–H and Supplementary Figure 1). These results indicated that theanine improved salt stress tolerance of the new shoots of tea plants.

### Exogenous Theanine Reduced Reactive Oxygen Species Accumulation in the Salt Stressed Leaves

**FIGURE 5:**
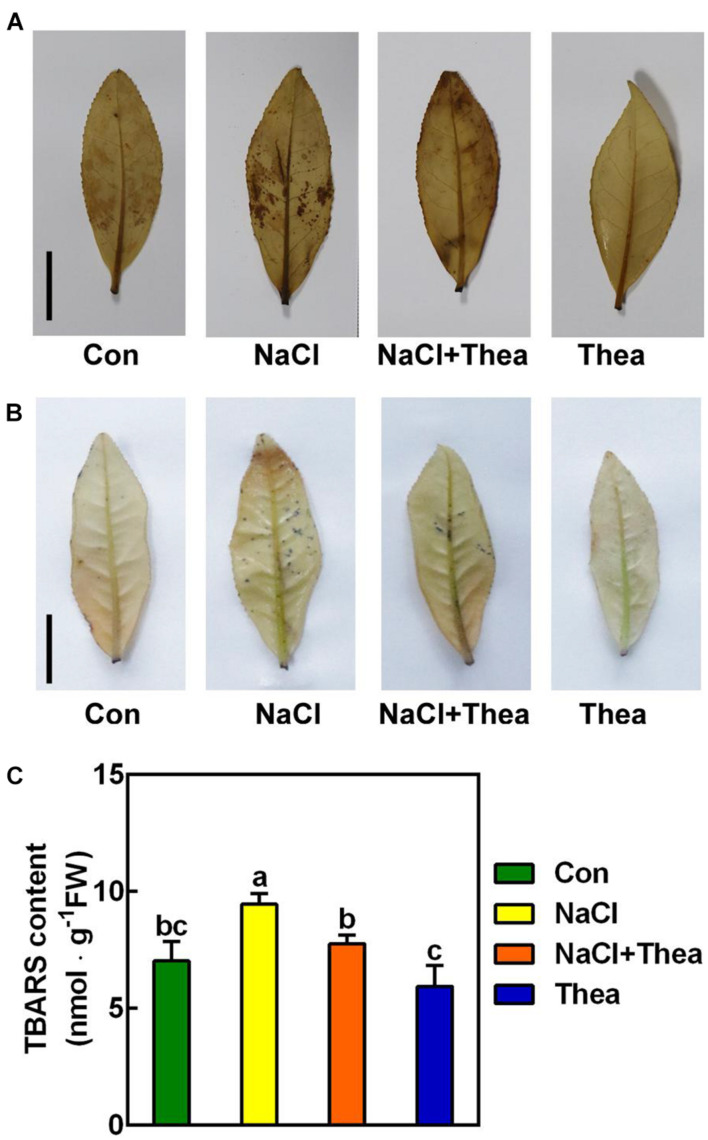
The effects of exogenous theanine on reactive oxygen species (ROS) accumulation in the leaves of new shoot exposed to salt stress. The new shoots of tea plants were treated in nutrient solution containing 150 mM NaCl (NaCl), 10 mM theanine (Thea), 150 mM NaCl, and 10 mM Thea (NaCl + Thea), with normal nutrient solution as control (Con). **(A,B)** H_2_O_2_ **(A)** and ${\text{O}}_{2}^{-•}$ **(B)** accumulation in the 2nd leaves of new shoots exposed to Con, NaCl, NaCl + Thea, and Thea. Three days after treatment, the leaves were stained with DAB and NBT, respectively, and were immediately photographed. Scale bar = 2 cm. **(C)** Thiobarbituric acid-reactive substances (TBARS; an indicator of lipid peroxidation) contents in the 2nd leaves. The contents were measured 3 days after treatment. At least three individual samples were analyzed. Data are means ± SE of at least three bilogical replicates. Bars with different letters are significantly different at *P* < 0.05 according to Duncan’s multiple range test.

Reactive oxygen species are induced by salt stress, which leads to growth stunt and even cell death (Considine and Foyer, 2021). To explore the mechanism underlying the improved salt stress tolerance by theanine, we examined the effects of theanine on ROS levels. By histochemical staining with diaminobenzidine (DAB) and nitroblue tetrazolium (NBT) staining, the accumulation of H_2_O_2_ and ${\text{O}}_{2}^{-•}$ in the 2nd leaves were, respectively, analyzed. As expected, compared to the shoots under control condition, NaCl-treated leaves accumulated more H_2_O_2_ and ${\text{O}}_{2}^{-•}$, while NaCl and theanine co-treatment greatly reduced H_2_O_2_ and ${\text{O}}_{2}^{-•}$ accumulation (Figures 5A,B). Consistently, theaine also reduced thiobarbituric acid-reactive substances (TBARS; an indicator of lipid peroxidation) accumulation in the salt stressed leaves (Figure 5C). These results suggested that theanine alleviated salt stress-induced oxidative damage to new shoots of tea plants.

**FIGURE 6:**
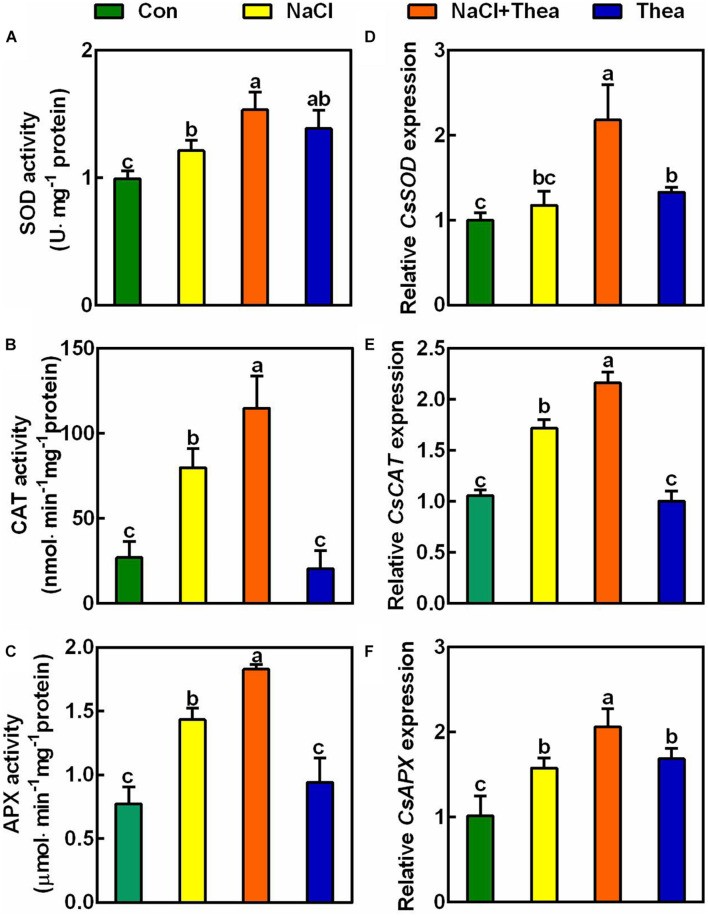
The effects of exogenous theanine on the activities of ROS scavenging enzymes and the expression of genes encoding the enzymes. The new shoots of tea plants were treated for 3 days in nutrient solution containing 150 mM NaCl (NaCl), 10 mM theanine (Thea), 150 mM NaCl and 10 mM Thea (NaCl + Thea), with normal nutrient solution as control (Con). **(A–C)** The activities of SOD, CAT, and APX in the 2nd leaves of the new shoots. **(D–F)** The expression of *CsSOD* (TEA00629), *CsCAT* (TEA002986), and *CsAPX* (TEA000543) in the 2nd leaves. The expression was analyzed 2 days after treatment by qRT-PCR. Expression levels were presented as values relative to Con samples, after normalization to the internal control gene *CsGAPDH* (TEA025584). Data are means ± SE of three biological replicates. Bars with different letters are significantly different at *P* < 0.05 according to Duncan’s multiple range test.

To explore how exogenous theanine reduced ROS accumulation, we examined the activities of ROS scavenging enzymes, including SOD, CAT, and APX. The results showed that salt stress increased SOD, CAT, and APX activities, and exogenous theanine further improved the activities of these enzymes (Figures 6A–C). At the level of gene expression, salt stress also significantly induced the expression of *CsCAT* and *CsAPX*, and exogenous theanine further significantly induced the expression of *CsSOD*, *CsCAT*, and *CsAPX* (Figures 6D–F). Therefore, exogenous theanine induced the expression of genes encoding ROS scavenging enzymes and increased the activities of these enzymes, under salt stress condition. These results supported the notion that salt stress-induced theanine contributes to salt stress tolerance *via* (at least partially) ROS scavenging pathway.

### Genetic Evidence Revealed That Catalase Is Required for Theanine-Induced Salinity Tolerance

**FIGURE 7:**
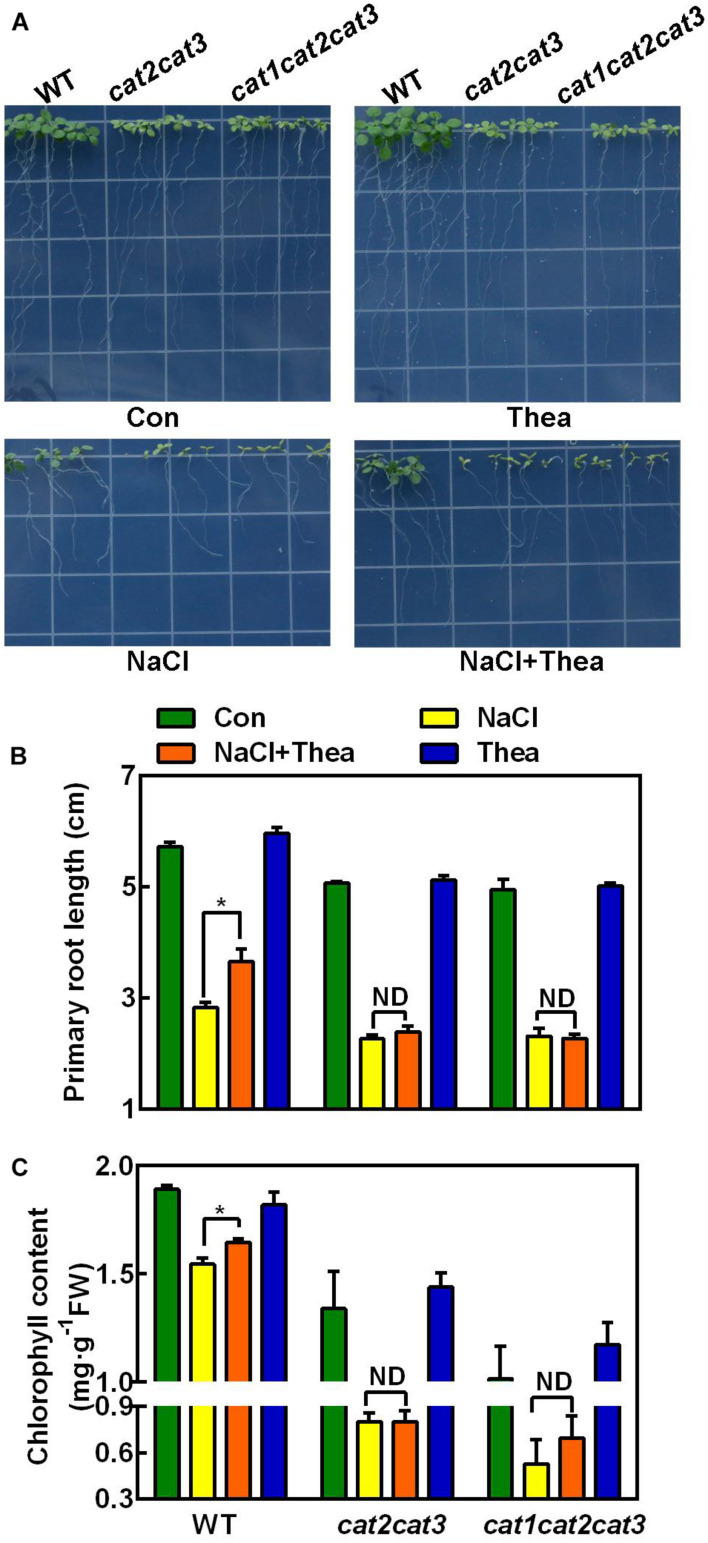
The effects of exogenous theanine on the salt stress tolerance of *Arabidopsis* wild-type (WT), *cat2cat3* and *cat1cat2cat3* mutants. **(A)** Phenotypes of WT *Arabidopsis* and *cat2cat3* and *cat1cat2cat3* mutants grown on MS medium containing 100 mM NaCl (NaCl), 100 mM NaCl and 1 mM theanine (NaCl + Thea), 1 mM theanine (Thea) for 10 days. The medium without NaCl and theanine was the control (Con). **(B,C)** Primary root length and total chlorophyll content in leaves of WT, *cat2cat3* and *cat1cat2cat3* mutants grown under the indicated conditions. Data are means ± SE of three biological replicates. Asterisks indicate significant differences from WT under NaCl treatment based on Student’s *t*-test (*P < 0.05). ND, no significant difference.

Catalases are highly responsive to stresses and are critical for scavenging H_2_O_2_ in plants (Mhamdi et al., 2012; Su et al., 2018). There are three genes *CAT1*, *CAT2*, and *CAT3* encoding catalases in Arabidopsis. *cat2cat3* double mutant and *cat1cat2cat3* triple mutant were obtained in Arabidopsis (Su et al., 2018). To investigate the role of ROS scavenging pathway in theanine-induced salt stress tolerance, we used these double and triple mutants for further study, given that we cannot knock down or overexpress the *CsCATs* in tea plants. Similar to the wild-type (WT), *cat2cat3* and *cat1cat2cat3* mutants also exhibited hypersensitivity to NaCl treatment (Figure 7). However, unlike WT, theanine did not improve salt stress tolerance of *cat2cat3* and *cat1cat2cat3* mutants, in terms of primary root growth and chlorophyll content (Figures 7A–C). These results indicated that catalase-mediated H_2_O_2_ scavenging pathway is required for theanine-induced salt stress tolerance in Arabidopsis, and implied that salt stress-induced theanine improves salt stress tolerance by modulating H_2_O_2_ scavenging in tea plants.

## Discussion

Theanine has favorable physiological effects on human health, including antioxidant and immune-regulation properties (Deng et al., 2016; Gong et al., 2019). A positive correlation exists between theanine and antioxidative activity, suggesting that theanine plays a role in modulating redox homeostasis in animals (Li et al., 2012; Deng et al., 2016; Zeng et al., 2020). Previous results showed that theanine contents in tea plants were influenced by abiotic stress, such as salinity, drought, and heat stress (Deng et al., 2012; Wang et al., 2016; Li et al., 2018a). It is known that these abiotic stresses, especially salt stress, elicits the accumulation of ROS and results in redox imbalance (Bose et al., 2013; Considine and Foyer, 2021).

Under our experimental conditions, theanine accumulation was obviously induced by salt stress in the new shoots of tea plants (Figure 2B). Besides, salt stress improved the glutamate accumulation, a direct substrate for theanine biosynthesis, as well as the alanine, a precursor of ethylamine in tea plants (Figures 2C,D; Sasaoka and Kito, 1964; Takeo, 1974). These findings are consistent with the former result (Deng et al., 2012). We further showed that the induced expression of theanine biosynthetic genes *CsAlaDC*, *CsGOGAT1*, *CsGOGAT2*, and *CsTSI* could be responsible for the increased theanine biosynthesis and accumulation.

Salt stress significantly inhibits electron transport at the oxidizing side of photosystem II (PSII) and decreases the PSII activity, which leads to reduced photosynthesis (Xia et al., 2004). A decline in chlorophyll content can also decrease the photosynthesis in plant during salinity stress (Heuer and Plaut, 1989). Thereby, the growth and productivity of plants were severely affected by salt stress (Jamil et al., 2014). Interestingly, in this study, we found the application of theanine was able to relieve high salinity-induced damage to the new shoots, the reduction of maximum quantum efficiency of photosystem II (Fv/Fm) and chlorophyll content (Figures 4A–D). Additionally, the induction of salt stress-responsive genes including *CsRD22*, *CsDREB2C*, *CsDREB1*, and *CsTSI* was also significantly recovered by theanine (Figures 4E–H). Combined with the protective effect of theanine in model plant Arabidopsis wild-type seedlings against salt stress (Supplementary Figure 1), and the high abundance of theanine in tea plants, we speculate that theanine is a key regulator of salt stress resistance in tea plants.

Redox imbalance, such as the overproduction of ROS and lipid peroxidation, was induced by salt stress (Miller et al., 2010; Considine and Foyer, 2021). ROS, in turn, inhibit protein synthesis and damage the protein subunits and pigments of PSII (Murata et al., 2007). Previous studies strongly revealed that an efficient antioxidant system contributes to salt stress tolerance in plants (Miller et al., 2010). The health benefits of theanine in animals are associated with its antioxidant actions (Williams et al., 2019). The present study demonstrated that theanine counteracted NaCl-induced H_2_O_2_, ${\text{O}}_{2}^{-•}$ and TBARS accumulation in the leaves of new shoots (Figure 5), and also increased the activities of SOD, CAT, and APX and the expression of the corresponding genes (Figures 6A–F). These results were consistent with the observations in animals. For example, theanine prevented ethanol-triggered ROS and MDA generation *via* restoring the antioxidant capability of hepatocytes (Li et al., 2012). The anti-inflammatory and antioxidative actions triggered by theanine were further confirmed in *Escherichia coli*-infected and D-galactose-induced liver dysfunction (Deng et al., 2016; Zeng et al., 2020). Together, our results suggested that theanine modulates oxidative damage in tea plants under salt stress condition.

Among the antioxidant enzymes, catalase is an important respiratory enzyme in plants, and its activity is closely related to plant resistance (Mhamdi et al., 2012). Chinese cabbage plants overexpressing maize catalase (ZmCAT) showed enhanced tolerance to high salinity (Tseng et al., 2007). Silencing *GhWRKY46* in cotton by virus-induced gene silencing reduced the catalase activity, and resulted in hypersensitivity to salt stress (Li et al., 2021). It was also found that Arabidopsis *cat2-1* mutant exhibited increased sensitivity to salt stress (Bueso et al., 2007). In the present study, we also found Arabidopsis *cat2cat3* double mutant and *cat1cat2cat3* triple mutant were hypersensitive to salt stress, and theanine could not enhance the salt stress tolerance of these mutants (Figure 7). This result provided genetic evidence that CAT-mediated redox homeostasis is required for theanine-induced salt stress tolerance.

Production of ROS was accelerated by over-reduced ferredoxin during photosynthetic electron transfer in the chloroplasts, impaired electron transport processes in the mitochondria and photorespiration in peroxisomes in plants (Ahmed et al., 2009). The nucleus-encoding ROS scavenging enzymes are responsible for maintaining the redox homeostasis in these organelles. In the present study, we showed that theanine application induced the expression of genes encoding SOD, APX, and CAT (Figures 6E–F). However, it is unclear how theanine induced the expression of these genes. Recently, the retrograde signaling from chloroplast to nucleus was reported to be critical for salt stress tolerance in plants (Park and Seo, 2021; Zhuang et al., 2021). This retrograde signaling is required for the induction of ROS scavenging pathway genes such as *CSD2* (Zhuang et al., 2021). Interestingly, theanine is mainly distributed in cytosol and chloroplast in the new shoots of tea plants (Fu et al., 2021). It will be interesting to investigate whether theanine regulates the expression of *SOD*, *APX*, and CAT *via* chloroplast to nucleus retrograde signaling pathway.

**FIGURE 8:**
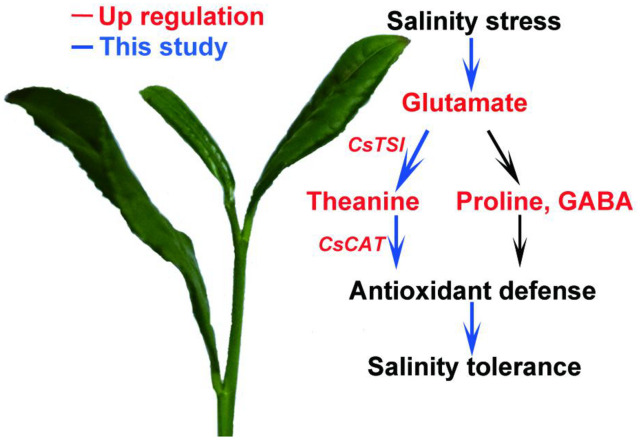
A proposed model of theanine-mediated tolerance to salt stress in tea plants. Salt stress increases glutamate accumulation and the expression of theanine biosynthetic genes to promote theanine biosynthesis. Theanine enhances plant high-salinity tolerance through a CAT-dependent ROS scavenging pathway.

Many studies have reported that proline or GABA improve salt stress tolerance in plants (Moukhtari et al., 2020; Wu et al., 2020). In this study, we showed that salt stress induces theanine biosynthesis, and the induced theanine is associated with enhanced salt stress tolerance in the new shoots of tea plants. Theanine-induced salt stress tolerance is probably *via* modulating ROS homeostasis in a CAT-dependent ROS scavenging pathway (Figure 8). To our knowledge, this is the first report to link theanine with salt stress tolerance and ROS homeostasis in tea plants. This study provided new insights into the physiological role of theanine in tea plants.

## Data Availability Statement

The original contributions presented in the study are included in the article/Supplementary Material, further inquiries can be directed to the corresponding author/s.

## Author Contributions

ZZ and ZC conceived the study and designed the experiments. ZC, SL, JL, and TC performed the experiments. QG and TY participated in the preparation of plant materials. ZC wrote the manuscript. ZZ revised and finalized the manuscript. All authors read and approved the final version of the manuscript.

## Conflict of Interest

The authors declare that the research was conducted in the absence of any commercial or financial relationships that could be construed as a potential conflict of interest.

## Publisher’s Note

All claims expressed in this article are solely those of the authors and do not necessarily represent those of their affiliated organizations, or those of the publisher, the editors and the reviewers. Any product that may be evaluated in this article, or claim that may be made by its manufacturer, is not guaranteed or endorsed by the publisher.
