# Supplementary material for: Theanine Improves Salt Stress Tolerance via Modulating Redox Homeostasis in Tea Plants (Camellia sinensis L.)
Source: Front Plant Sci. 2021 Oct 15;12:770398. doi: 10.3389/fpls.2021.770398 (PMC8554060; doi:10.3389/fpls.2021.770398)
Supplement: Supplementary file 1 [file Data_Sheet_1.docx]

**Supplementary**
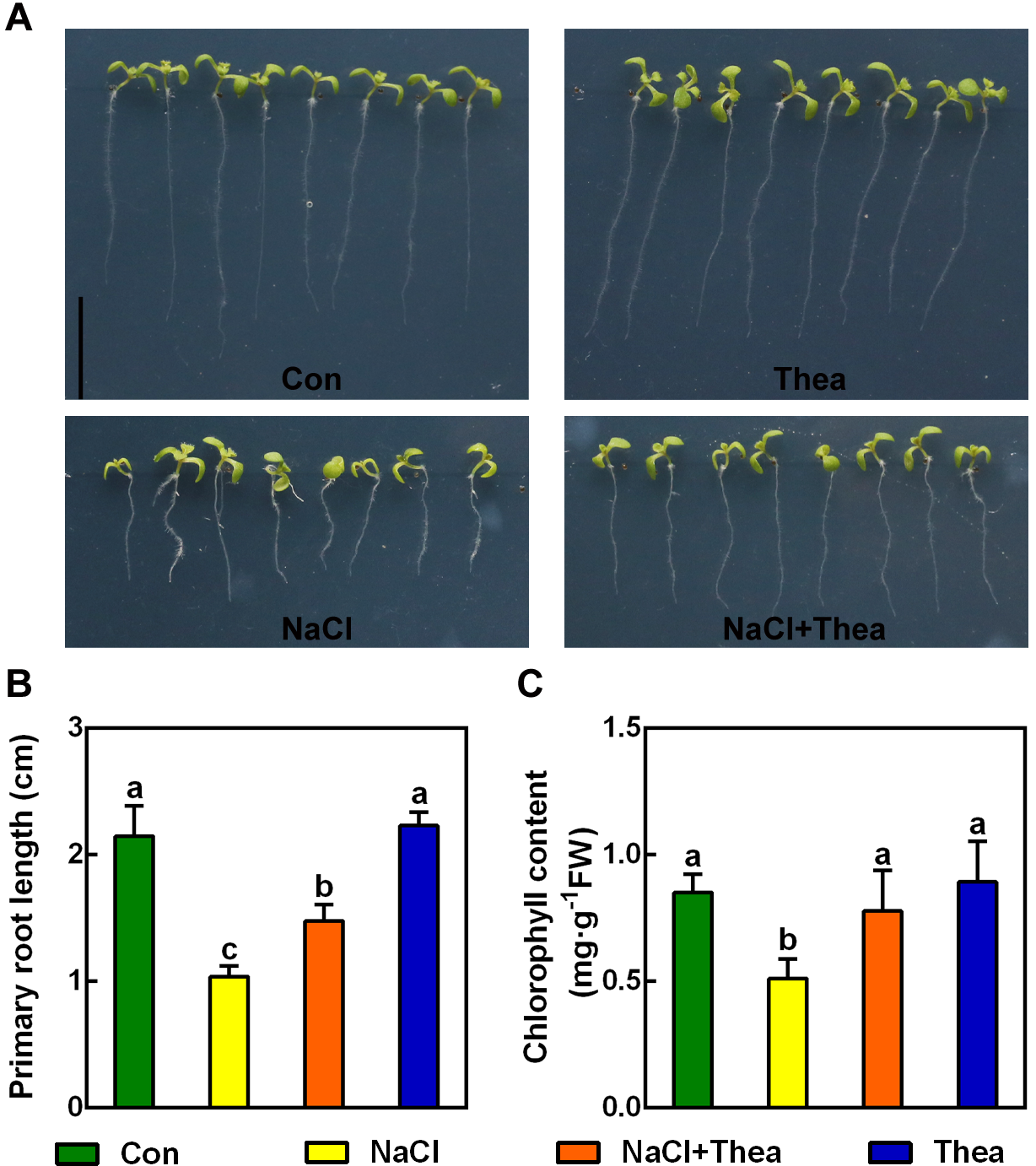
 **Figure 1** Theanine-mediated salt stress tolerance in *Arabidopsis*. **(A)** Phenotypes of wild-type *Arabidopsis* grown on MS medium containing 125 mM NaCl (NaCl), 125 mM NaCl and 1 mM theanine (NaCl + Thea), 1 mM theanine (Thea), and 0 mM NaCl and 0 mM theanine (Con). **(B-C)** Primary root length and total chlorophyll contents in leaves of the seedlings grown under the Con, NaCl, NaCl + Thea and Thea conditions. Scale bar = 1 cm. Data are means ± SE of three biological replicates. Bars with different letters denote significant difference at *P* <0.05 according to Duncan’s multiple range test.

**Supplementary Table 1** The sequences of primers used for qRT-PCR.

| Primer name | Gene ID | Sequences (5’→3’) |
| --- | --- | --- |
| *CsGAPDH-F* | *TEA025584* | TTGGCATCGTTGAGGGTCT |
| *CsGAPDH-R* |  | CAGTGGGAACACGGAAAGC |
| *CsTSI-F* | *TEA015198* | GTTGATGTTTCTGGGCAGCA |
| *CsTSI-R* |  | CTCACCCACACCAGTCAGAT |
| *CsGSII-1.1-F* | *TEA015580* | GTGGAGAGCCAATCCCAACA |
| *CsGSII-1.1-R* |  | ACCAATCGGCCACTTCACTT |
| *CsGSII-1.2-F* | *TEA032123* | AGGAAAGCACGAAACAGCTG |
| *CsGSII-1.2-R* |  | TGGAAGTGACAACGTACGGA |
| *CsGSII-1.3-F* | *TEA032217* | ATGAATTGTGGGTGGCTCGT |
| *CsGSII-1.3-R* |  | AACCCCCATCACTCCTCGTA |
| *CsGSII-2-F* | *TEA028194* | GTCGCTAATCGCGGTTGTTC |
| *CsGSII-2-R* |  | AGCCAATAAGGCCGTCACAA |
| *CsGOGAT-1-F* | *TEA003892* | TGCCAAGGGCAAGAAGGTAG |
| *CsGOGAT-1-R* |  | TCCACACGGAATATGCGAGG |
| *CsGOGAT-2-F* | *TEA026779* | GGGGGCCTTATGATGTACGG |
| *CsGOGAT-2-R* |  | TCCCGTCCAGGTACAGGTAG |
| *CsAlaDC-F* | *TEA005658* | CACTGTGATGGGGCTCTGTT |
| *CsAlaDC-R* |  | TGTTATCTGGACACCGCACG |
| *CsRD22-F* | *TEA005584* | GCGGCTACTTTCTTACCTCGCCA |
| *CsRD22-R* |  | GTTTCTTTTTGTACCTCCGTGGA |
| *CsDREB2C-F* | *TEA000861* | GAGTGATGGTCATTTCCTGAATC |
| *CsDREB2C-R* |  | TATCATAGTTTCCCTCTTGCTGT |
| *CsDREB1-F* | *TEA010806* | TGTAATGACAATACGGGGTCCA |
| *CsDREB1-R* |  | AGCAAGGAGGTGGTGAAAGC |
| *CsSOD-F* | *TEA006329* | AGTGTGCCACGCTCTTTGTCAT |
| *CsSOD-R* |  | ATCTCGTTGAGGATTCTTCAC |
| *CsCAT-F* | *TEA002986* | AGGTATGATTCGGTTCGCCA |
| *CsCAT-R* |  | CGCTCTTGCCTGTCTGGTG |
| *CsAPX-F* | *TEA000543* | TCCAAATGCTACTAAAGGG |
| *CsAPX-R* |  | CAGCTCCACAAAGAACGAA |
